# Sex differences in HIV-1 persistence and the implications for a cure

**DOI:** 10.3389/fgwh.2022.942345

**Published:** 2022-09-23

**Authors:** Shringar Rao

**Affiliations:** Department of Biochemistry, Erasmus University Medical Centre, Rotterdam, Netherlands

**Keywords:** HIV-1 cure, biological sex, viral latency, viral reservoirs, sex-specific differences

## Abstract

Of the 38 million people currently living with Human Immunodeficiency Virus type-1 (HIV-1), women, especially adolescents and young women, are disproportionally affected by the HIV-1 pandemic. Acquired immunodeficiency syndrome (AIDS) - related illnesses are the leading cause of death in women of reproductive age worldwide. Although combination antiretroviral therapy (cART) can suppress viral replication, cART is not curative due to the presence of a long-lived viral reservoir that persists despite treatment. Biological sex influences the characteristics of the viral reservoir as well as the immune responses to infection, factors that can have a significant impact on the design and quantification of HIV-1 curative interventions in which women are grossly underrepresented. This mini-review will provide an update on the current understanding of the impact of biological sex on the viral reservoir and will discuss the implications of these differences in the context of the development of potential HIV-1 curative strategies, with a focus on the shock and kill approach to an HIV-1 cure. This mini-review will also highlight the current gaps in the knowledge of sex-based differences in HIV-1 persistence and will speculate on approaches to address them to promote the development of more scalable, effective curative approaches for people living with HIV-1.

## Introduction–The need for an HIV-1 cure

More than half of the 38 million people worldwide living with Human Immunodeficiency Virus Type-1 (HIV-1) are women. Combination antiretroviral therapy (cART) to control HIV-1 is lifesaving, but not curative due to the establishment of a persistent, latent, viral reservoir that is not eliminated by cART. cART must therefore be taken lifelong and people living with HIV-1 (PLWH) are burdened with detrimental side effects, social stigma, costs and the possibility of developing drug resistance. Importantly, more than one in four PLWH are currently not on cART, rendering them vulnerable to acquired immunodeficiency syndrome (AIDS)-related complications that could otherwise be prevented by effective cART. Women, in particular, are disproportionally affected by HIV-1 with high burdens of comorbidities (1) and this is reflected in the fact that the leading cause of death among women of reproductive age worldwide is AIDS-related illnesses (2). Despite women accounting for 52% of PLWH worldwide, women are grossly underrepresented in HIV-1 curative clinical trials (3), with only 11% of HIV-1 cure trial participants between 1991 and 2011 being women (4). Therefore, there remains a dire need for the development of scalable HIV-1 curative therapies to control the pandemic (5).

Both the biological attribute of sex and the socio-cultural factor of gender are critical variables to consider in biomedical research, and they have also been reported to influence HIV-1 transmission, pathogenesis and response to treatment [reviewed in Scully (6, 7)]. Although so far under-investigated, understanding the biological sex-specific differences in HIV-1 infection is critical for the development of therapies that are efficacious in both sexes, especially in the quest for an HIV-1 cure. This mini-review briefly discusses what is currently understood about sex-specific differences in HIV-1 persistence and the host immune responses to control the viral reservoir, with a focus on cis-women with an XX sex chromosome living with HIV-1 (herein referred to as WLHIV). These differences will then be analyzed in the context of the development of an HIV-1 cure using a shock and kill approach by laying out the outstanding questions in the field and by speculating on approaches that account for sex-specific differences in HIV-1 infection.

## Sex differences in the viral reservoir

**Figure 1:**
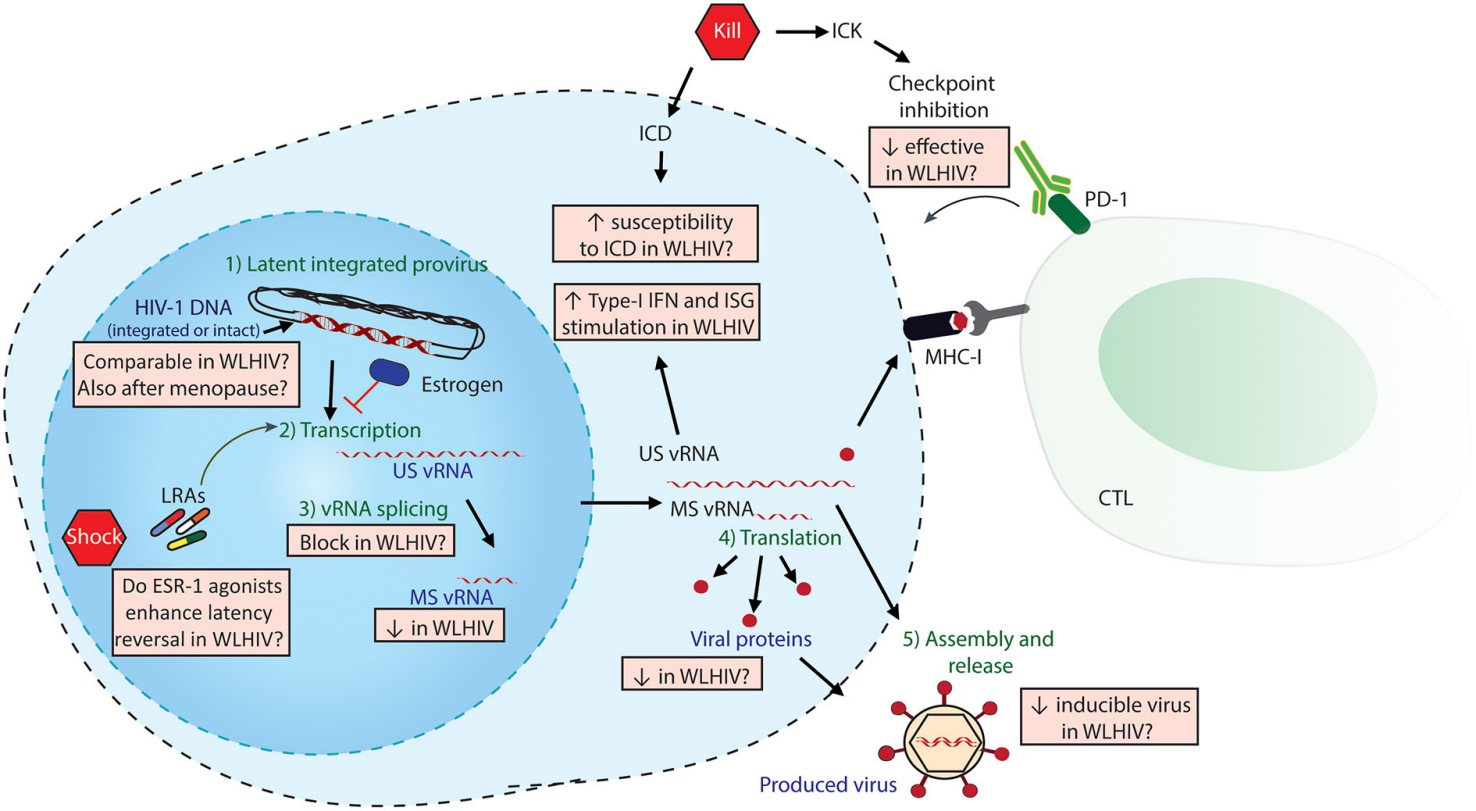
Sex-specific differences in HIV-1 Shock and Kill. The latent HIV-1 provirus needs to undergo multiple steps (in green) to effectively produce virus. Viral quantitation techniques measure different stages of latency reversal (in purple), including HIV-DNA, cell-associated HIV-1 vRNAs (both unspliced US and multiply spliced MS) and released viral protein or vRNA. The steps of the shock and kill approach are indicated in red pentagons: latency reversing agents (LRAs) to reactivate the provirus, followed by either induced cell death (ICD) or immune-mediated killing (ICK) strategies. ICK strategies may include checkpoint inhibition to enhance cytotoxic T-cell lymphocyte (CTL)-mediated killing. The multiple sex-specific differences, both reported and speculated, are depicted in orange boxes.

The main obstacle to an HIV-1 cure is the establishment of a viral reservoir by HIV-1 upon infection that persists during cART. HIV-1 curative strategies are aimed at either eliminating or reducing the viral reservoir, or permanently silencing it, thereby abrogating the need for lifelong cART (5, 8). An outstanding question in HIV-1 cure research has been the development of a standardized technique to measure the viral reservoir (8). To date, multiple techniques have been used, each with its advantages and limitations (8). These techniques measure different stages of the latent virus and its reactivation from latency, including cell-associated HIV-1 DNA (total, integrated or intact), cell-associated viral RNA (both unspliced-US and multiply spliced-MS), viral proteins expression and the inducibility/replication-competence of viral RNAs (vRNAs) or proteins following latency reversal. Figure 1 depicts different stages of latency reversal and indicates sex-specific differences observed, as discussed below.

### Are cell-associated HIV-1 DNA levels comparable between sexes?

Two early cross-sectional studies in ART-treated PLWH (*n* = 243, 28% women) (9) and (*n* = 522, 29% women) (10) that aimed to characterize the factors associated with a smaller viral reservoir by measuring total HIV-1 DNA found that WLHIV were more likely to have lower HIV-1 DNA levels than men. In a longitudinal study with 101 ART-treated donors (21% women), WLHIV also had lower levels of HIV-1 DNA, although the results did not reach statistical significance (11). In seminal work by Scully et al., sex differences in viral reservoir size and activity were specifically studied in a cohort of 84 well-matched ART suppressed men and WLHIV (49% women) (12). They observed comparable levels of total or integrated HIV-1 DNA between the sexes (12). Three later studies also observed no significant differences in total, integrated or intact HIV-1 DNA in men compared to WLHIV (13–15). This discrepancy could be because the earliest two studies (9, 10) were measuring HIV-1 DNA in whole peripheral blood mononuclear cells (PBMCs), rather than in isolated CD4+ T-cells and women have a different lymphocyte percentage in whole PBMCs before and after menopausal transition (16).

One longitudinal study also addressed sex-specific differences in reservoir dynamics during aging using more than 400 samples collected longitudinally from 90 donors (56% WLHIV) (17). They found that although both men and women have a significant decline in total HIV-1 DNA over time, WLHIV reservoirs are more dynamic and have a significantly slower rate of decline than men, a difference that becomes more pronounced with age (17). The onset of menopause can cause alterations in immune responses and viral replication in WLHIV [reviewed in Abelman and Tien (18)], which could contribute to this difference seen in slower reservoir decay in aging women. Work by the same group showed that in a sample size of 50 women, premenopausal women have lower levels of HIV-1 DNA than post-menopausal women (15), implying that some loss of viral control is observed after the onset of menopause, thereby resulting in an increase in the size of the viral reservoir. However, this study measured HIV-1 DNA from whole PBMCs and post-menopausal women have a higher percentage of leukocytes in PBMCs than pre-menopausal women (16), which may also explain this effect observed. An outstanding question in the field that remains is whether there is indeed a difference in reservoir size between post and pre-menopausal women when quantifying total, integrated or intact HIV-1 DNA from isolated CD4+ T-cells.

### What is the significance of apparent lower MS-VRNA levels in WLHIV?

In a study by Scully et al. to assess sex-specific differences in the HIV-1 reservoir, no differences in cell-associated US-vRNA were observed between WLHIV and men. However, they reported that WLHIV have a six-fold lower expression of cell-associated MS-vRNA than men (12). This study also measured the inducibility of the viral reservoir by using TILDA, a reservoir quantification tool that measures the frequency of the induction of MS-vRNA transcripts. When normalized to the levels of integrated HIV-1 DNA, WLHIV had a statistically insignificant yet considerable two-fold decrease in the inducibility of the MS-vRNA transcripts compared to men (12). Another study by Gianelli et al. also reported that in a cohort of 99 PLWH (51% women), cell-associated MS-vRNA could be detected in only 54% of WLHIV, but it could be detected in all the male participants of the study (15). The production of MS-vRNA is associated with viral production and higher plasma vRNA levels (19) and the balance of MS-vRNA to US-vRNAs ratio can have implications for the maintenance of viral latency [discussed in Pasternak and Berkhout (20)]. Lower expression of MS-vRNA could explain why WLHIV have lower levels of plasma viral RNA and residual viremia during cART than men (21–23), but this needs to be validated with further research. It also remains to be assessed if sex-specific differences in alternative splicing (24, 25), differences in pharmacokinetics and pharmacodynamics of cART in WLHIV (26) or increased immune clearance of MS-vRNA-producing cells in WLHIV are responsible for lower cell-associated MS-vRNA levels in WLHIV. Other areas that warrant further investigation are to understand what the clinical implications of the apparent lower MS-vRNA levels in WLHIV are, whether this correlates to better immune control and the lower HIV-1 plasma levels observed in WLHIV (22), whether lower MS-vRNA levels in WLHIV result in less viral protein (p24) production upon restimulation and what the implications of this are in the readouts of the efficacy of HIV-1 curative interventions in clinical trials.

### Do WLHIV have lesser inducible viral reservoirs than men?

Some reservoir quantitation techniques like the quantitative viral outgrowth assay (qVOA) measure the induction of replication-competent virus by measuring released viral proteins or vRNA in the produced virus. Using this technique, two groups describe conflicting reports on the effect of sex on the inducible viral reservoir (13, 14). In one study in Rakai, Uganda (n=90, 63% women), WLHIV had a lower replication-competent virus than men. In contrast, another report (*n* = 61, 36% women) could not find any differences in the frequency of cells harboring replication-competent virus as measured both by qVOA or by quantifying the intact HIV-1 DNA (14). These differences could be due to sampling size or other sources of variation such as the HIV-1 subtype since the study in Uganda had participants with predominantly D or A HIV-1 subtypes compared to the predominantly B HIV-1 subtypes present in the West where the other study was conducted. Further investigation is necessary to assess if WLHIV indeed have lower inducible reservoirs than men, whether the lower MS-vRNA observed in women contributes to these differences and whether sex-specific differences are comparable across different HIV-1 subtypes.

## Sex differences in shock and kill

One of the most widely investigated strategies for an HIV-1 cure is known as the “Shock and Kill” approach which employs the use of latency-reversing agents (LRAs) to reactivate the viral reservoir followed by the use of pharmacological or immunomodulatory interventions to eliminate the reactivated infected cells (5). How sex-specific differences may influence the application of this strategy is discussed below and depicted in Figure 1.

### Shock: How latency reversal may work differently for WLHIV

Post-integration HIV-1 latency is controlled by multiple epigenetic mechanisms like nucleosome positioning, histone modifications, DNA methylations or silencing activities mediated by non-coding RNAs [reviewed in Verdikt et al. (27)]. Most latency-reversing agents currently described target these stages epigenetic mechanisms to induce latency reversal. Sex-differences in DNA-methylation patterns have been reported in whole blood and brain samples (28, 29). Sex-differences in chromatin accessibility and histone modifications have also been reported (30, 31). Therefore, it is important to include participants of both sexes when assessing latency reversal agents that target epigenetic mechanisms. These differences could also be relevant when considering the “block and lock” approach to an HIV-1 cure involves permanently silencing the HIV-1 provirus by inducing silencing of the 5'-LTR of the virus.

Although latency was earlier speculated to be due to an inhibition of transcription initiation, it has recently come to light that latency is also regulated at post-transcriptional levels like vRNA splicing (32, 33). Although commonly used LRAs induce US-vRNA production, they do not induce robust MS-vRNA indicating a block at splicing (19, 32, 34), possibly contributing to their limited clinical efficacy in reducing the size of the viral reservoir. WLHIV produce lower levels of cell-associated MS-vRNA and are suggested to have lesser inducible viral reservoirs (12, 13). Therefore, the vRNA splicing block observed upon LRA treatment may be more pronounced in WLHIV, resulting in suboptimal latency reversal with lesser released viral proteins as a marker of the inducible viral reservoir. The development of LRAs that also modulate vRNA splicing may promote more robust latency reversal in WLHIV. Given the differences in MS-vRNA production in WLHIV and the clinical significance of MS-vRNA production (19), it is important that any studies, pre-clinical or clinical, that quantify latency reversal include a measurement of MS-vRNA as well as US-vRNA and then stratify their findings based on sex.

The increased viral control in WLHIV is also attributed to the findings that estrogen can inhibit HIV-1 transcription (35, 36) and can control viral replication also during acute HIV-1 infection (37). In untreated HIV-1 infection, plasma viral loads dropped as levels of β-estradiol increased in menstruating women (38). In a longitudinal study in cells from WLHIV, incrementally increasing levels of inducible MS-vRNA cells were observed during reproductive aging, as biologically active estrogen levels declined (17), thereby further implicating estrogen in being able to control HIV-1 viral production. Estrogen was shown to repress HIV-1 promoter activity by inducing the formation of a complex containing β-catenin that binds the HIV- LTR (36). In an unbiased shRNA screen, the estrogen receptor ESR-1 was found to be a negative regulator of viral reactivation by accumulating on the HIV-1 LTR, with ESR-1 agonists reinforcing HIV-1 latency and antagonists reactivating HIV-1 (39). In a primary CD4+ T-cell model of HIV-1 latency, ESR-1 agonists like tamoxifen were also shown to synergise with known LRAs like SAHA or TNF-α (39). In a cohort of 12 matched donors (50% women), latency reversal in cells from WLHIV by T-cell receptor engagement resulted in lower production of MS-vRNA than in males. The addition of ESR-1 agonists further decreased MS-vRNA induction in cells from both sexes with more robust effects in cells from WLHIV (39), a finding also later observed by Scully et al. (12). Conversely, a combination of an ESR-1 antagonist with SAHA resulted in a more robust latency reversal in cells from WLHIV than in those from men (39). Follow up work resulted in a clinical trial with 31 post-menopausal WLHIV to assess if tamoxifen and SAHA treatment could result in enhanced latency reversal *in vivo* than SAHA alone (40). No synergistic activity was observed in co-treated participants, but this could also be alluded to the modest latency reversal by SAHA, the postmenopausal status of participants and low levels of vRNA expression below limits of detection result. This study however was the first ever HIV-1 cure clinical trial to be conducted exclusively in women, and further work is necessary to address how latency reversal can be influenced by sex hormones like estrogen. This is also very relevant for trans-women living with HIV-1 who may be on gender-affirming hormone therapy. Since most of the studies on the effect of estradiol on latency reversal were conducted using subtype-B HIV-1, it still remains to be assessed if similar effects of estradiol are observed in non-B HIV-1 subtypes that have distinct LTRs. This may have implications for latency-reversal approaches and it may be possible that women require an additional intervention to antagonize ESR-1 to sensitize them for more effective latency reversal.

### Kill: How do we target the viral reservoir in WLHIV?

The second aspect of the shock and kill approach involves the clearance of the reactivated cells by pharmacological or immunomodulatory interventions that depend on the host immune response, of which biological sex is an important determinant (41), also in the context of HIV-1 (7). One approach to clear the reservoir is by pharmacologically inducing cell death (ICD) by activating the host innate immune pathways to trigger selective cell death of infected cells (42); as we have shown with the use of inhibitors of DEAD-box helicase 3X (DDX3) that induced the production of pro-apoptotic Type-I interferons (IFNs) in vRNA-expressing cells resulting in their selective cell death and a reduction in the size of the inducible reservoir (43). Women are reported to produce higher levels of IFN-α upon toll-like receptor (TLR)7 stimulation as compared to men (44), mediated by the stimulatory effect of estrogen on plasmacytoid dendritic cells (pDCs) function (45). This is also observed in HIV-1 infection where higher levels of TLR-7-induced IFN-α and Interferon-stimulated genes (ISGs) production have been reported in WLHIV (14, 46, 47). TLR7 agonists are highly investigated in HIV-1 cure approaches due to their latency reversal and immunomodulatory effect [reviewed in Martinsen et al. (48)], and their use may result in different effects in WLHIV that need to be considered and reported in clinical trials. Given that IFNs and ISGs are key mediators of apoptosis (49), also in the context of viral infections (43, 50), it remains to be studied if ICD approaches to target the viral reservoir may demonstrate sex-specific differences due to enhanced IFN-α production in WLHIV. Another outstanding question is if the apparent splicing block of vRNAs in WLHIV leads to the accumulation of intron-containing US-vRNA that is a potent activator of innate immune signaling (51, 52), thereby resulting in increased innate immune responses in WLHIV.

An alternative approach to clear the viral reservoir is to stimulate infected cell killing (ICK) by enhancing humoral or cell-mediated immune responses with broadly neutralizing antibodies, therapeutic vaccinations, engineered T-cells or other immunomodulatory agents to eliminate reactivating cells (53). Immune exhaustion is prevalent during chronic HIV-1 infection, with increased expression of immune checkpoint PD-1 on infected cells (54). Lower levels of PD-1 were reported in CD4+ and CD8+ T-cells from WLHIV (12) and the size of the inducible viral reservoir was positively correlated with PD-1 expression in men, but not in WLHIV (13). Another study did not observe any difference in PD-1 expression in WLHIV and men (14), but differences in sample size number and participant characteristics could explain this apparent lack of phenotype. Immune checkpoint inhibitors are widely explored as immunomodulators in controlling HIV-1 [reviewed in Castelli et al. (55)], and with the reported sex-specific differences in immune checkpoint marker expression in WLHIV, the use of these inhibitors needs to be specifically evaluated in WLHIV. Although sex-specific differences in broadly-neutralizing antibody production and HIV-1 vaccine responses have not been explicitly reported, the sex-specific differences in general immune responses (41) warrant further attention to evaluating ICK in HIV-1 cure trials.

## Reduce and control for WLHIV

In the HIV-1 cure field, studies indicate that the complete elimination of the viral reservoir is unlikely to be achieved with the current approaches. Therefore, shock and kill can be used to reduce the size of the reservoir, not eliminate it, followed by approaches to enhance post-ART viral control, a strategy known collectively as “reduce and control” providing a functional cure for HIV-1 (8). Effective viral control is observed in “elite controllers” who can spontaneously control viremia or “post-treatment controllers” who have sustained viral suppression after interruption of ART (56, 57). In one case report, a WLHIV who demonstrated elite control of the virus had no detectable provirus in >1.5 billion cells, indicating a possibility of spontaneous viral clearance (58). Pharmacologically reinforcing the splicing block observed in WLHIV who have lower levels of MS-vRNA could also lead to enhanced viral control. Some studies demonstrate that WLHIV are more likely to be elite controllers than men (crude odds ratio, 2.35; 95% confidence interval (CI), 1.63–3.37) (59, 60). These reports indicate that the “reduce and control” approach may also demonstrate sex-specific differences that can be exploited.

## Outstanding questions and what we can do

An HIV-1 cure has been elusive in the last 40 years of living with the virus, and sex-specific differences in HIV-1 biology may further occlude the development of a scalable cure effective in WLHIV. This mini-review has laid out some key areas of investigation in context of the shock and kill HIV-1 cure approach, but multiple questions remain to understand HIV-1 persistence in WLHIV and how this may influence a cure. For example, it is still not understood if the viral reservoir in WLHIV is harbored in the same anatomical locations as in men or if the provirus is contained in the same CD4+ T-cell subsets. Although this mini-review is focussed on cis-women, HIV-1 cure studies also need to include transgender individuals since they are disproportionally burdened by HIV-1 (61). HIV-1 cure clinical trials need to be designed to ensure sufficient representation of WLHIV and their participation must be actively encouraged. This is critically important given the demonstrated differences in pharmacokinetics and pharmacodynamics in women (26), also in response to antiretroviral treatment (62), that could affect drug delivery in WLHIV. Gene therapy is also a widely pursued approach to an HIV-1 cure [reviewed in Peterson and Kiem (63)] and although no sex differences in *in vitro* efficacy of gene editing strategies have been reported, *in vivo* delivery of these therapies may be require a different approach in WLHIV because of the distinct pharmacokinetic profiles of women. Time to viral rebound followed by analytical treatment interruption is a common endpoint in HIV-1 cure trials, and since women are better at controlling viral replication, they may take longer to viral rebound (64). Moreover, since viral reservoirs may be less inducible in WLHIV and produce less MS-vRNA, trial design must take into account these differences by using appropriate clinical endpoints. Sex-specific differences must also be accounted for in pre-clinical HIV-1 research. Studies using cell lines should report the sex of the cell line used since this can also influence results (65). When using primary cells *in vitro* models of HIV-1 latency and *ex vivo* cells from PLWH in pre-clinical, the sex of the donors need to be taken into account when analyzing the results. Finally, in all pre-clinical and clinical HIV-1 cure research that measure latency reversal, immune activation or reservoir depletion, sex stratification and reporting of data will contribute to filling in the many gaps in understanding HIV-1 persistence in WLHIV. Only if we can effectively treat WLHIV, can we control the current global pandemic.

## Author contributions

SR conducted to the review and writing of the manuscript.

## Funding

This work is supported by grants from the Dutch Aidsfonds (P-53302) and from the Gilead Research Scholars award to SR.

## Conflict of interest

The author declares that the research was conducted in the absence of any commercial or financial relationships that could be construed as a potential conflict of interest.

## Publisher's note

All claims expressed in this article are solely those of the authors and do not necessarily represent those of their affiliated organizations, or those of the publisher, the editors and the reviewers. Any product that may be evaluated in this article, or claim that may be made by its manufacturer, is not guaranteed or endorsed by the publisher.

## References

[1] Raffe S Sabin C Gilleece Y Women against viruses in Europe EACS. comorbidities in women living with HIV: a systematic review. HIV Med. (2022) 23:331–61. 10.1111/hiv.13240 35243744 PMC9311813

[2] WHO. Global Health Estimates: Cause-specific Mortality, 2000–2019. Available online at: https://www.who.int/data/gho/data/themes/mortality-and-global-health-estimates/ghe-leading-causes-of-death (accessed May 12, 2022).

[3] Curno MJ Rossi S Hodges-Mameletzis I Johnston R Price MA Heidari S. A systematic review of the inclusion (or Exclusion) of women in HIV Research: From Clinical Studies of Antiretrovirals and Vaccines to Cure Strategies. J Acquir Immune Defic Syndr. (2016) 71:181–8. 10.1097/QAI.0000000000000842 26361171

[4] Johnston RE Heitzeg MM. Sex, age, race and intervention type in clinical studies of HIV cure: a systematic review. AIDS Res Hum Retroviruses. (2015) 31:85–97. 10.1089/aid.2014.0205 25313793 PMC4287187

[5] Ndung'u T McCune JM Deeks SG. Why and where an HIV cure is needed and how it might be achieved. Nature. (2019) 576:397–405. 10.1038/s41586-019-1841-8 31853080 PMC8052635

[6] Scully EP. Sex differences in HIV infection. Curr HIV/AIDS Rep. (2018) 15:136–46. 10.1007/s11904-018-0383-2 29504062 PMC5882769

[7] Scully EP. Sex differences in HIV infection: mystique vs. machismo. Pathog Immun. (2018) 3:82–113. 10.20411/pai.v3i1.238 30140783 PMC6103226

[8] Deeks SG Archin N Cannon P Collins S Jones RB de Jong MAWP. Research priorities for an HIV cure: international AIDS society global scientific strategy 2021. Nat Med. (2021) 27:2085–98. 10.1038/s41591-021-01590-5 34848888

[9] Fourati S Flandre P Calin R Carcelain G Soulie C Lambert-Niclot S. Factors associated with a low HIV reservoir in patients with prolonged suppressive antiretroviral therapy. J Antimicrob Chemother. (2014) 69:753–6. 10.1093/jac/dkt428 24187041

[10] Cuzin L Pugliese P Sauné K Allavena C Ghosn J Cottalorda J. Levels of intracellular HIV-DNA in patients with suppressive antiretroviral therapy. AIDS. (2015) 29:1665–71. 10.1097/QAD.0000000000000723 26372277

[11] Gandhi RT McMahon DK Bosch RJ Lalama CM Cyktor JC Macatangay BJ. Levels of HIV-1 persistence on antiretroviral therapy are not associated with markers of inflammation or activation. PLoS Pathog. (2017) 13:e1006285. 10.1371/journal.ppat.1006285 28426825 PMC5398724

[12] Scully EP Gandhi M Johnston R Hoh R Lockhart A Dobrowolski C. Sex-based differences in human immunodeficiency virus type 1 reservoir activity and residual immune activation. J Infect Dis. (2019) 219:1084–94. 10.1093/infdis/jiy617 30371873 PMC6784502

[13] Prodger JL Capoferri AA Yu K Lai J Reynolds SJ Kasule J. Reduced HIV-1 latent reservoir outgrowth and distinct immune correlates among women in Rakai, Uganda. JCI Insight. (2020) 5:e139287. 10.1172/jci.insight.139287 32544096 PMC7453892

[14] Falcinelli SD Shook-Sa BE Dewey MG Sridhar S Read J Kirchherr J. Impact of biological sex on immune activation and frequency of the latent HIV reservoir during suppressive antiretroviral therapy. J Infect Dis. (2020) 222:1843–52. 10.1093/infdis/jiaa298 32496542 PMC7653086

[15] Gianella S Tran SM Morris S Vargas M Porrachia M Oliveira MF. Sex differences in CMV replication and HIV persistence during suppressive ART. Open Forum Infect Dis. (2020) 7:ofaa289. 10.1093/ofid/ofaa289 32793766 PMC7415302

[16] Chen Y Zhang Y Zhao G Chen C Yang P Ye S. Difference in leukocyte composition between women before and after menopausal age, and distinct sexual dimorphism. PLoS ONE. (2016) 11:e0162953. 10.1371/journal.pone.0162953 27657912 PMC5033487

[17] Gianella S Rawlings SA Dobrowolski C Nakazawa M Chaillon A Strain M. Sex differences in human immunodeficiency virus persistence and reservoir size during aging. Clin Infect Dis. (2021) 75:73–80. 10.1093/cid/ciab873 34612493 PMC9402699

[18] Abelman R Tien PC. The Reproductive transition: effects on viral replication, immune activation, and metabolism in women with HIV infection. Curr HIV/AIDS Rep. (2022) 19:133–9. 10.1007/s11904-021-00594-7 34878617 PMC8904361

[19] Zerbato JM Khoury G Zhao W Gartner MJ Pascoe RD Rhodes A. Multiply spliced HIV RNA is a predictive measure of virus production *ex vivo* and *in vivo* following reversal of HIV latency. EBioMedicine. (2021) 65:103241. 10.1016/j.ebiom.2021.103241 33647768 PMC7920823

[20] Pasternak AO Berkhout B. The splice of life: does RNA processing have a role in HIV-1 persistence? Viruses. (2021) 13:1751. 10.3390/v13091751 34578332 PMC8471011

[21] Farzadegan H Hoover DR Astemborski J Lyles CM Margolick JB Markham RB. Sex differences in HIV-1 viral load and progression to AIDS. Lancet. (1998) 352:1510–4. 10.1016/S0140-6736(98)02372-1 9820299

[22] Sterling TR Vlahov D Astemborski J Hoover DR Margolick JB Quinn TC. Initial plasma HIV-1 RNA levels and progression to AIDS in women and men. N Engl J Med. (2001) 344:720–5. 10.1056/NEJM200103083441003 11236775

[23] Cyktor JC Bosch RJ Mar H Macatangay BJ Collier AC Hogg E. Association of male sex and obesity with residual plasma human immunodeficiency virus 1 viremia in persons on long-term antiretroviral therapy. J Infect Dis. (2021) 223:462–70. 10.1093/infdis/jiaa373 32603416 PMC7881329

[24] Karlebach G Veiga DFT Mays AD Chatzipantsiou C Barja PP Chatzou M. The impact of biological sex on alternative splicing. bioRxiv [Preprint]. (2020) 490904. 10.1101/490904

[25] Trabzuni D Ramasamy A Imran S Walker R Smith C Weale ME. Widespread sex differences in gene expression and splicing in the adult human brain. Nat Commun. (2013) 4:2771. 10.1038/ncomms3771 24264146 PMC3868224

[26] Gandhi M Aweeka F Greenblatt RM Blaschke TF. Sex differences in pharmacokinetics and pharmacodynamics. Annu Rev Pharmacol Toxicol. (2004) 44:499–523. 10.1146/annurev.pharmtox.44.101802.121453 14744256

[27] Verdikt R Hernalsteens O Van Lint C. Epigenetic mechanisms of HIV-1 persistence. Vaccines (Basel). (2021) 9:514. 10.3390/vaccines9050514 34067608 PMC8156729

[28] Grant OA Wang Y Kumari M Zabet NR Schalkwyk L. Characterising sex differences of autosomal DNA methylation in whole blood using the Illumina EPIC array. Clin Epigenetics. (2022) 14:62. 10.1186/s13148-022-01279-7 35568878 PMC9107695

[29] Xia Y Dai R Wang K Jiao C Zhang C Xu Y. Sex-differential DNA methylation and associated regulation networks in human brain implicated in the sex-biased risks of psychiatric disorders. Mol Psychiatry. (2021) 26:835–48. 10.1038/s41380-019-0416-2 30976086 PMC6788945

[30] Sugathan A Waxman DJ. Genome-wide analysis of chromatin states reveals distinct mechanisms of sex-dependent gene regulation in male and female mouse liver. Mol Cell Biol. (2013) 33:3594–610. 10.1128/MCB.00280-13 23836885 PMC3753870

[31] Ratnu VS Emami MR Bredy TW. Genetic and epigenetic factors underlying sex differences in the regulation of gene expression in the brain. J Neurosci Res. (2017) 95:301–10. 10.1002/jnr.23886 27870402 PMC5120607

[32] Yukl SA Kaiser P Kim P Telwatte S Joshi SK Vu M. HIV latency in isolated patient CD4(+) T cells may be due to blocks in HIV transcriptional elongation, completion, and splicing. Sci Transl Med. (2018) 10:eaap9927. 10.1126/scitranslmed.aap9927 29491188 PMC5959841

[33] Moron-Lopez S Telwatte S Sarabia I Battivelli E Montano M Macedo AB. Human splice factors contribute to latent HIV infection in primary cell models and blood CD4+ T cells from ART-treated individuals. PLoS Pathog. (2020) 16:e1009060. 10.1371/journal.ppat.1009060 33253324 PMC7728277

[34] Mota TM McCann CD Danesh A Huang SH Magat DB Ren Y. Integrated Assessment of Viral Transcription, Antigen Presentation, and CD8(+) T Cell Function Reveals Multiple Limitations of Class I-Selective Histone Deacetylase Inhibitors during HIV-1 Latency Reversal. J Virol. (2020) 94:e01845–19. 10.1128/JVI.01845-19 32051267 PMC7163115

[35] Asin SN Heimberg AM Eszterhas SK Rollenhagen C Howell AL. Estradiol and progesterone regulate HIV type 1 replication in peripheral blood cells. AIDS Res Hum Retroviruses. (2008) 24:701–16. 10.1089/aid.2007.0108 18462082

[36] Szotek EL Narasipura SD Al-Harthi L. 17β-Estradiol inhibits HIV-1 by inducing a complex formation between β-catenin and estrogen receptor α on the HIV promoter to suppress HIV transcription. Virology. (2013) 443:375–83. 10.1016/j.virol.2013.05.027 23769242 PMC3722310

[37] El-Badry E Macharia G Claiborne D Brooks K Dilernia DA Goepfert P. Better Viral Control despite Higher CD4(+) T Cell Activation during Acute HIV-1 Infection in Zambian Women Is Linked to the Sex Hormone Estradiol. J Virol. (2020) 94:e00758–20. 10.1128/JVI.00758-20 32461316 PMC7394904

[38] Greenblatt RM Ameli N Grant RM Bacchetti P Taylor RN. Impact of the ovulatory cycle on virologic and immunologic markers in HIV-infected women. J Infect Dis. (2000) 181:82–90. 10.1086/315207 10608754

[39] Das B Dobrowolski C Luttge B Valadkhan S Chomont N Johnston R. Estrogen receptor-1 is a key regulator of HIV-1 latency that imparts gender-specific restrictions on the latent reservoir. Proc Nat Acad Sci. (2018) 115:E7795–E804. 10.1073/pnas.1803468115 30061382 PMC6099847

[40] Scully EP Aga E Tsibris A Archin N Starr K Ma Q. Impact of tamoxifen on vorinostat-induced human immunodeficiency virus expression in women on antiretroviral therapy: AIDS clinical trials group A5366, The MOXIE Trial. Clin Infect Dis. (2022). 10.1093/cid/ciac136. [Epub ahead of print]. 35176755 PMC9555843

[41] Klein SL Flanagan KL. Sex differences in immune responses. Nat Rev Immunol. (2016) 16:626–38. 10.1038/nri.2016.90 27546235

[42] Chen M Li M Budai MM Rice AP Kimata JT Mohan M. Clearance of HIV-1 or SIV reservoirs by promotion of apoptosis and inhibition of autophagy: Targeting intracellular molecules in cure-directed strategies. J Leukoc Biol. (2022). 10.1002/JLB.4MR0222-606. [Epub ahead of print]. 35362118 PMC9522917

[43] Rao S Lungu C Crespo R Steijaert TH Gorska A Palstra RJ. Selective cell death in HIV-1-infected cells by DDX3 inhibitors leads to depletion of the inducible reservoir. Nat Commun. (2021) 12:2475. 10.1038/s41467-021-22608-z 33931637 PMC8087668

[44] Berghöfer B Frommer T Haley G Fink L Bein G Hackstein H. TLR7 ligands induce higher IFN-alpha production in females. J Immunol. (2006) 177:2088–96. 10.4049/jimmunol.177.4.2088 16887967

[45] Seillet C Laffont S Trémollières F Rouquié N Ribot C Arnal JF. The TLR-mediated response of plasmacytoid dendritic cells is positively regulated by estradiol in vivo through cell-intrinsic estrogen receptor α signaling. Blood. (2012) 119:454–64. 10.1182/blood-2011-08-371831 22096248

[46] Meier A Chang JJ Chan ES Pollard RB Sidhu HK Kulkarni S. Sex differences in the Toll-like receptor-mediated response of plasmacytoid dendritic cells to HIV-1. Nat Med. (2009) 15:955–9. 10.1038/nm.2004 19597505 PMC2821111

[47] Chang JJ Woods M Lindsay RJ Doyle EH Griesbeck M Chan ES. Higher expression of several interferon-stimulated genes in HIV-1-infected females after adjusting for the level of viral replication. J Infect Dis. (2013) 208:830–8. 10.1093/infdis/jit262 23757341 PMC3733517

[48] Martinsen JT Gunst JD Højen JF Tolstrup M Søgaard OS. The use of toll-like receptor agonists in HIV-1 cure strategies. Front Immunol. (2020) 11:1112. 10.3389/fimmu.2020.01112 32595636 PMC7300204

[49] Chawla-Sarkar M Lindner DJ Liu YF Williams BR Sen GC Silverman RH. Apoptosis and interferons: role of interferon-stimulated genes as mediators of apoptosis. Apoptosis. (2003) 8:237–49. 10.1023/A:1023668705040 12766484

[50] Balachandran S Roberts PC Kipperman T Bhalla KN Compans RW Archer DR. Alpha/beta interferons potentiate virus-induced apoptosis through activation of the FADD/Caspase-8 death signaling pathway. J Virol. (2000) 74:1513–23. 10.1128/JVI.74.3.1513-1523.2000 10627563 PMC111487

[51] McCauley SM Kim K Nowosielska A Dauphin A Yurkovetskiy L Diehl WE. Intron-containing RNA from the HIV-1 provirus activates type I interferon and inflammatory cytokines. Nat Commun. (2018) 9:5305. 10.1038/s41467-018-07753-2 30546110 PMC6294009

[52] Akiyama H Miller CM Ettinger CR Belkina AC Snyder-Cappione JE Gummuluru S. HIV-1 intron-containing RNA expression induces innate immune activation and T cell dysfunction. Nat Commun. (2018) 9:3450. 10.1038/s41467-018-05899-7 30150664 PMC6110775

[53] Lee MJ Fidler S Frater J. Immunotherapeutic approaches to HIV cure and remission. Curr Opin Infect Dis. (2022) 35:31–41. 10.1097/QCO.0000000000000803 34873077

[54] Breton G Chomont N Takata H Fromentin R Ahlers J Filali-Mouhim A. Programmed death-1 is a marker for abnormal distribution of naive/memory T cell subsets in HIV-1 infection. J Immunol. (2013) 191:2194–204. 10.4049/jimmunol.1200646 23918986 PMC3815464

[55] Castelli V Lombardi A Palomba E Bozzi G Ungaro R Alagna L. Immune checkpoint inhibitors in people living with HIV/AIDS: Facts and controversies. Cells. (2021) 10:2227. 10.3390/cells10092227 34571876 PMC8467545

[56] Blankson JN. Control of HIV-1 replication in elite suppressors. Discov Med. (2010) 9:261–6. 20350494

[57] Sáez-Cirión A Bacchus C Hocqueloux L Avettand-Fenoel V Girault I Lecuroux C. Post-treatment HIV-1 controllers with a long-term virological remission after the interruption of early initiated antiretroviral therapy ANRS VISCONTI study. PLoS Pathog. (2013) 9:e1003211. 10.1371/journal.ppat.1003211 23516360 PMC3597518

[58] Turk G Seiger K Lian X Sun W Parsons EM Gao C. A possible sterilizing cure of HIV-1 infection without stem cell transplantation. Ann Intern Med. (2021) 175:95–100. 10.7326/L21-0297 34781719 PMC9215120

[59] Crowell TA Gebo KA Blankson JN Korthuis PT Yehia BR Rutstein RM. Hospitalization rates and reasons among HIV elite controllers and persons with medically controlled HIV infection. J Infect Dis. (2015) 211:1692–702. 10.1093/infdis/jiu809 25512624 PMC4447832

[60] Madec Y Boufassa F Porter K Meyer L Collaboration C. Spontaneous control of viral load and CD4 cell count progression among HIV-1 seroconverters. AIDS. (2005) 19:2001–7. 10.1097/01.aids.0000194134.28135.cd 16260907

[61] Stutterheim SE van Dijk M Wang H Jonas KJ. The worldwide burden of HIV in transgender individuals: An updated systematic review and meta-analysis. PLoS ONE. (2021) 16:e0260063. 10.1371/journal.pone.0260063 34851961 PMC8635361

[62] Ofotokun I Pomeroy C. Sex differences in adverse reactions to antiretroviral drugs. Top HIV Med. (2003) 11:55–9. 12717043

[63] Peterson CW Kiem HP. Cell and gene therapy for HIV cure. Curr Top Microbiol Immunol. (2018) 417:211–48. 10.1007/82_2017_71 29256135

[64] Le CN Britto P Brummel SS Hoffman RM Li JZ Flynn PM. Time to viral rebound and safety after antiretroviral treatment interruption in postpartum women compared with men. AIDS. (2019) 33:2149–56. 10.1097/QAD.0000000000002334 31373919 PMC6832824

[65] Shah K McCormack CE Bradbury NA. Do you know the sex of your cells? Am J Physiol Cell Physiol. (2014) 306:C3–18. 10.1152/ajpcell.00281.2013 24196532 PMC3919971

